# Increased abundance of proteobacteria in aggressive Crohn’s disease seven years after diagnosis

**DOI:** 10.1038/s41598-019-49833-3

**Published:** 2019-09-17

**Authors:** M. K. Vester-Andersen, H. C. Mirsepasi-Lauridsen, M. V. Prosberg, C. O. Mortensen, C. Träger, K. Skovsen, T. Thorkilgaard, C. Nøjgaard, I. Vind, K. A. Krogfelt, N. Sørensen, F. Bendtsen, A. M. Petersen

**Affiliations:** 1 Gastrounit, Hvidovre Hospital, University of Copenhagen, København, Denmark; 2 Department of Internal medicine, Zealand University Hospital, Køge, Denmark; 3 Department of Bacteria, Parasites and Fungi, Statens Serum Institut, Copenhagen, Denmark; 4 Department of Virus and Microbial Diagnostics, Statens Serum Institut, Copenhagen, Denmark; 5 Department of Gastroenterology, Herlev and Gentofte Hospital, University of Copenhagen, København, Denmark; 6 Clinical-Microbiomics, Ole Maaløesvej 3, Copenhagen, Denmark; 7 Department of Clinical Microbiology, Hvidovre Hospital, University of Copenhagen, København, Denmark

**Keywords:** Bacteria, Inflammatory bowel disease, Dysbiosis

## Abstract

Intestinal dysbiosis in inflammatory bowel disease (IBD) patients depend on disease activity. We aimed to characterize the microbiota after 7 years of follow-up in an unselected cohort of IBD patients according to disease activity and disease severity. Fifty eight Crohn’s disease (CD) and 82 ulcerative colitis (UC) patients were included. Disease activity was assessed by the Harvey-Bradshaw Index for CD and Simple Clinical Colitis Activity Index for UC. Microbiota diversity was assessed by 16S rDNA MiSeq sequencing. In UC patients with active disease and in CD patients with aggressive disease the richness (number of OTUs, p = 0.018 and p = 0.013, respectively) and diversity (Shannons index, p = 0.017 and p = 0.023, respectively) were significantly decreased. In the active UC group there was a significant decrease in abundance of the phylum Firmicutes (p = 0.018). The same was found in CD patients with aggressive disease (p = 0.05) while the abundance of Proteobacteria phylum showed a significant increase (p = 0.03) in CD patients. We found a change in the microbial abundance in UC patients with active disease and in CD patients with aggressive disease. These results suggest that dysbiosis of the gut in IBD patients is not only related to current activity but also to the course of the disease.

## Introduction

The potential role of the gut microbiota as a driver of the inflammatory process in inflammatory bowel diseases (IBD) has gained increasing attention in the past decades and the body of research in this field has expanded vastly. The aetiology of IBD is unknown but is generally believed to result from complex interactions between the immune system, the gut microbiota and environmental factors in genetic susceptible individuals^1^.

Several studies have found an imbalance in the gut microbiota in IBD patients compared to non-IBD controls with an overall loss of diversity, a depletion of firmicutes^2–7^ and an increase of Proteobacteria^8–11^. In Crohn’s disease (CD) the primary finding has been a decrease in abundance of *Faecalibacterium prausnitzii* (Firmicutes)^2,12–15^ a butyrate-producing bacteria as well as an increase in the adherent-invasive *E. coli* (Proteobacteria)^8,16–18^, the latter particularly found to be linked to ileal Crohn’s disease. Studies in ulcerative colitis (UC) have been less consistent^2,14,19–22^. Yet, we have previously shown that *E. coli* play an important role in IBD pathogenesis in UC patients^23–25^. However, fecal microbiota transplantation (FMT) appears to be effective for induction of remission in UC as described in a recent meta-analysis of four placebo controlled trials^26^. These findings do support that intestinal dysbiosis play an important role as trigger of inflammation also in UC.

The clinical course of IBD is characterized by periods of active inflammation superseded by periods of remission. The phenotypic appearance of both CD and UC influences the choice of treatment and prognosis. CD patients with ileal disease are at higher risk of treatment with systemic steroids^27^ and surgery^27–29^ during the first flare of the disease. A recent study suggests that IBD can be subdivided into three phenotypes as ileal CD, colonic CD and UC according to genetic composition^30^. Human studies on microbiota in IBD patients have shown a positive correlation between the NOD2 risk allele and the relative abundance of Enterobacteriaceae in intestinal specimens from IBD patients^31^ and the study by Gevers *et al*. demonstrated differences in the mucosa-associated microbiome of ileal and rectal biopsies^32^. Furthermore, the course of disease comprising periods of quiescent disease with alternating periods of active disease and thereby repeating or changing medication regimens may also influence gut microbiota^33–35^ Thus, it is possible that different IBD phenotypes as well as disease course are associated to different specific microbial characteristics.

The aim of our study was to investigate if the former described dysbiosis could be recovered in a cohort of unselected patients with CD and UC diagnosed in 2003–04 in Copenhagen, Denmark. The cohort is well described through 7 years of follow-up with a complete phenotypic characterization based on endoscopic findings, diagnostic procedures (MR/CT/US), medical treatment and surgical procedures^36^. Subsequently, we wished to characterize the microbiota according to disease activity state, disease severity and disease localization in the included population after 7 years of disease duration.

## Results

Fecal microbiota from 58 UC patients and 82 CD patients and 30 healthy controls was successfully determined by 16S rDNA sequencing.

### Diversity of the microbiota in relation to disease activity

**Figure 1:**
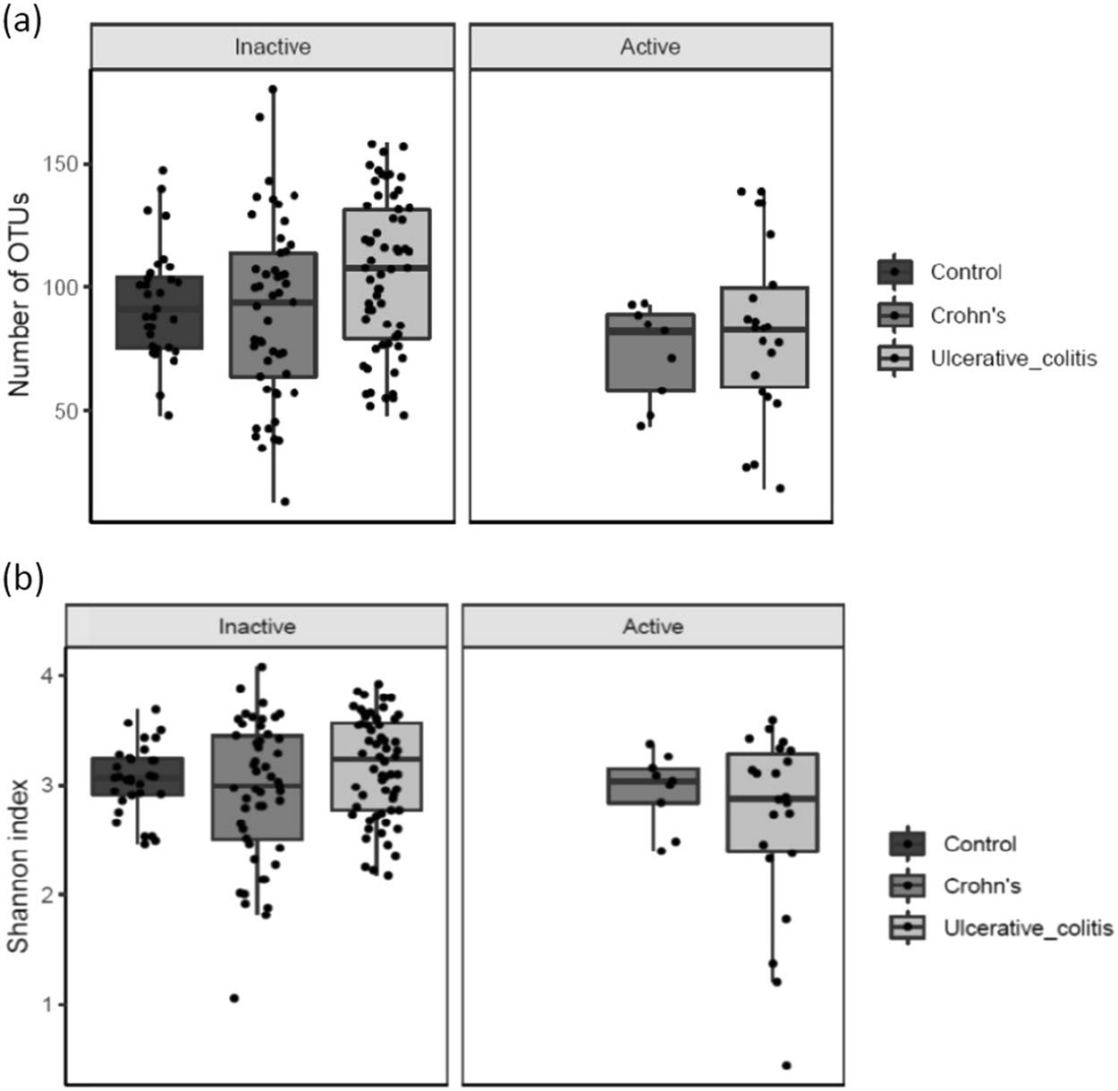
(**a**) (Number of OTU’s) and (**b**) (Shannons Index). Diversity of microbiota according to disease activity in ulcerative colitis and Crohn’s disease. NA indicates controls. Individual observations are marked as block black dots.

When comparing patients with active disease (HB-score ≥ 5/SCCAI score ≥ 3) to patients with inactive disease, the richness (number of OTUs, p = 0.018) (Fig. 1a) and diversity (Shannon index, p = 0.017) (Fig. 1b) was lower in active compared to inactive UC patients. There were no significant differences between active and inactive CD.

The microbiota diversity of inactive IBD was comparable to healthy controls.

### Phyla-level differences in abundance in relation to disease activity

**Figure 2:**
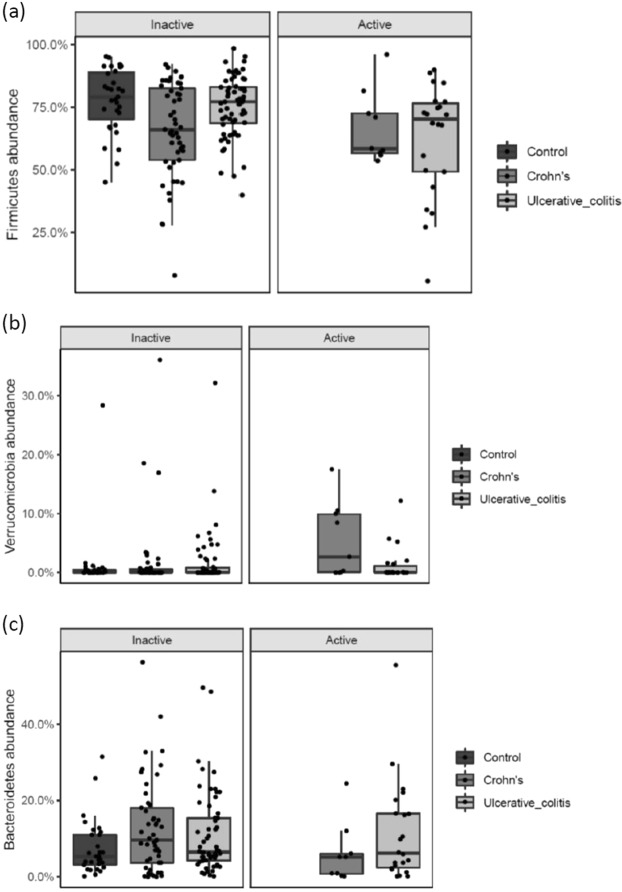
(**a**–**c**) Phyla-level differences in abundance according to disease activity state in UC and CD patients. NA indicates controls. Individual observations are marked as block black dots.

When looking into differences in abundance in patients with active versus inactive disease, there was a significantly lower abundance of Firmicutes (p = 0.018) in UC patients with active disease compared to patients with inactive disease. In CD, the abundance of Verrucomicrobia was significantly increased (p = 0.038) and a tendency for a lower abundance of Bacteroidetes (p = 0.071) in patients with active disease (Fig. 2a–c).

### Diversity and abundancy in relation to disease severity

**Figure 3:**
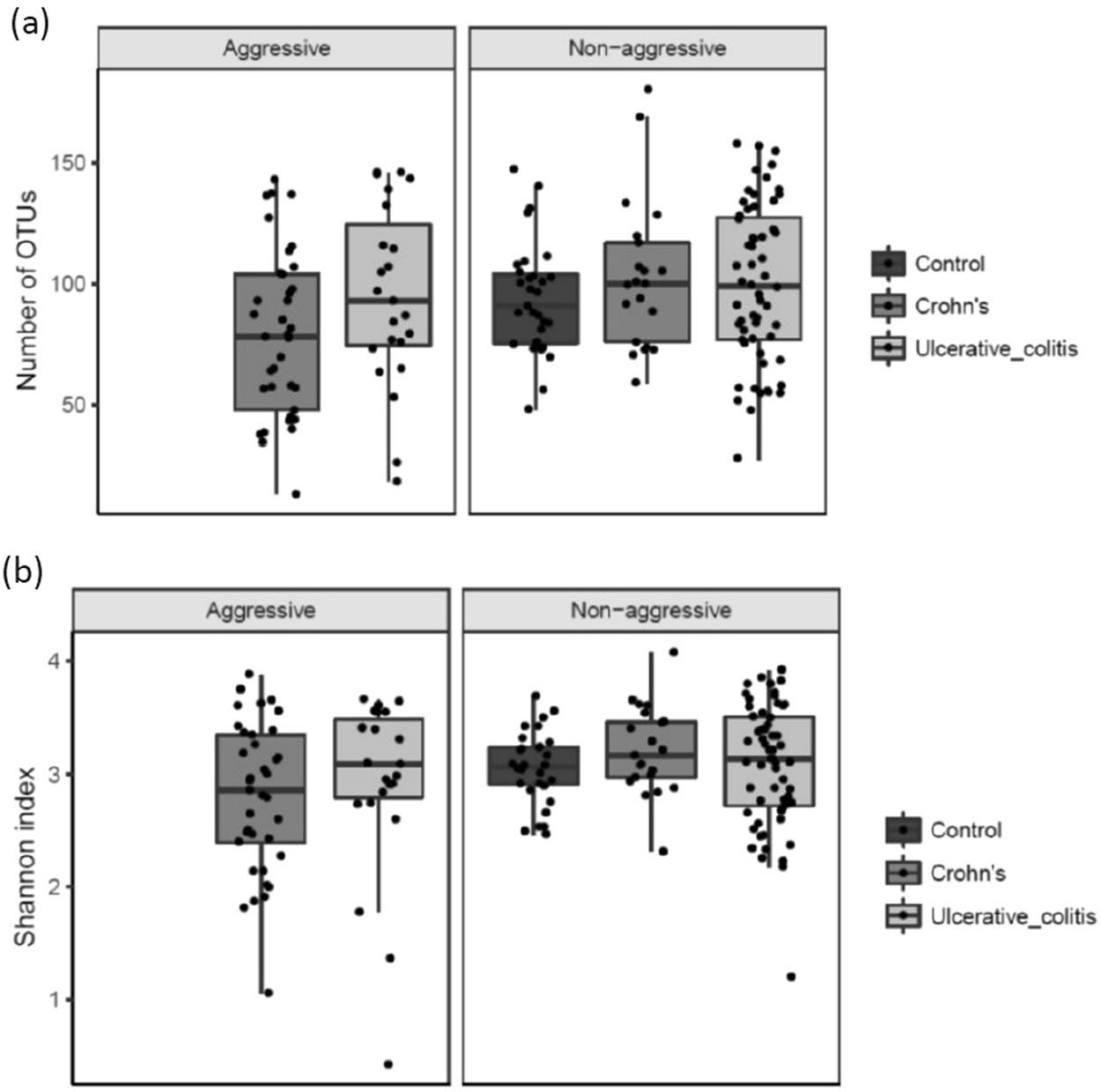
(**a**) (Number of OTU’s) and (**b**) (Shannons Index). Diversity of the microbiota according to disease severity. NA indicates controls. Individual observations are marked as block dots.

According to our definition of disease severity (with aggressive disease being defined as ≥3 courses of systemic steroids (≥50 mg/day) and/or biological therapy (any doses) and/or surgical resection during the 7 years of follow-up) a significant decrease of richness (number of OTUs, p = 0.013) and diversity (Shannon index, p = 0.023) in CD patients with an aggressive disease course was observed (Fig. 3a,b).

There was no change in richness and diversity observed between non-aggressive and aggressive disease for UC.

**Figure 4:**
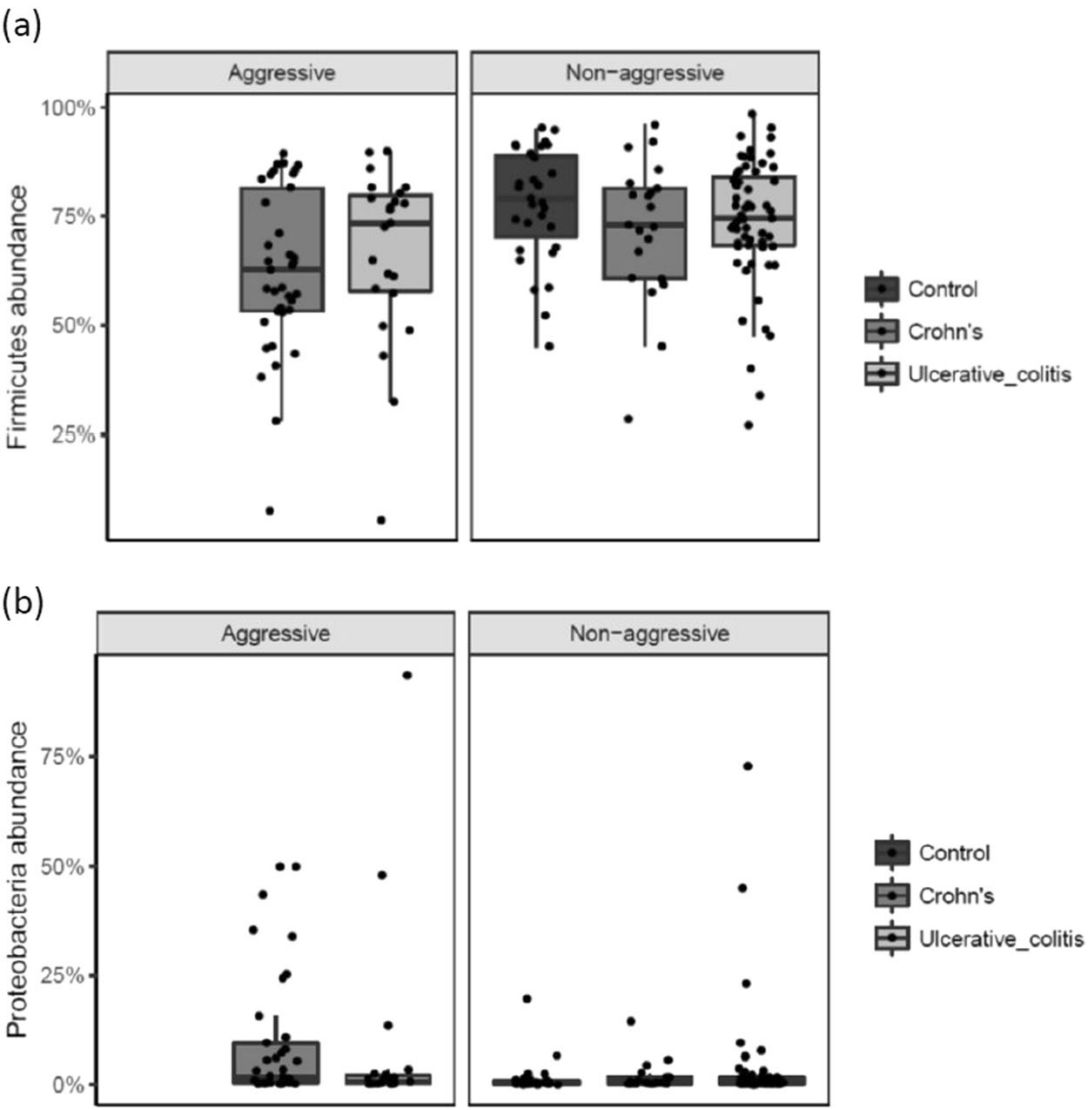
(**a**,**b**) Phyla-level differences in abundance according to disease severity in UC and CD patients. Individual observations are marked as block black dots.

At the phylum-level there was a significant difference in the sequence abundance of a phylum according to disease severity in CD patients. The abundance of Firmicutes showed a significant decrease (p = 0.05) and the abundance of Proteobacteria (p = 0.03) a significant increase in CD patients with aggressive disease compared to CD patients with non-aggressive disease (Fig. 4a,b). There were no significant findings in the phyla-abundance among UC patients with aggressive disease compared to non-aggressive disease.

### Diversity in patients with active disease in relation to extent/localization of disease

When looking at patients with active disease in relation to extent (UC) or localization (CD) of disease, we found no significant differences in richness and diversity (number of OTUs or Shannons Index) among either UC or CD patients.

### Phyla-level differences in abundance in patients with active disease in relation to disease extent/localization

When comparing patients according to phenotype, we found no differences in abundance among UC or CD patients with active disease compared to inactive disease according to localization of disease.

## Discussion

In this study, we wished to describe the microbiota of the gut in IBD patients after 7 years of disease duration and explore gut dysbiosis in an unselected group of IBD patients according to the course of disease. We found a significant difference in microbial diversity in UC patients with active disease compared to inactive disease, as well as in CD patients with aggressive disease compared to non-aggressive disease. The abundance of Firmicutes was decreased in UC patients with active disease and in CD patients with aggressive disease. Also, in CD patients with aggressive disease, the abundance of Proteobacteria was increased. However, phenotypic presentation of disease did not seem to influence the microbial community of the gut.

We found a significantly lower diversity overall in patients with clinical active UC, with the microbial diversity of patients with inactive disease being comparable to the one of healthy controls. When looking at the phylum level the abundance of Firmicutes was decreased in UC patients with active disease as also observed in CD patients with an aggressive disease course. This is in accordance to previous studies^14,19–21,37,38^. *F. praustnitzii* belong to the phylum Firmicutes. A low count of these bacteria has previously been found to be associated to the risk of relapse in UC patients, and the *F. praustnitzii* population recovers in patients who achieve remission^39^. The association between gut microbiota and disease activity was also found in a recent meta-analysis, supporting our results^40^. In CD, a decrease in the population of Firmicutes have been found to be associated with relapsing disease compared with non-relapsing disease after discontinuation of TNF-alpha inhibitor treatment^10^. The increased abundance of Proteobacteria in CD patients found in our study has previously, in other studies, on a genus level mainly shown to be driven by an increase in the *adherent-invasive E. coli* in CD patients^8,16,18^. Furthermore, it was shown that pathogenic *E. coli* B2 phylogroup were isolated from feces of UC patients^23–25^. It has previously been shown that patients with extensive and active UC (based on SCCAI-scores) have a significantly higher level of Proteobacteria in mucosal biopsies compared with patients with limited extend and less active disease^41^. Our data do not support these findings.

In our study, we investigated if the severity of disease over time influenced the microbial community in incident CD and UC patients. Inception cohorts are characterized by encompassing unselected patients who represent the entire span of disease activity from a mild to a severe disease course., We did not have baseline microbiome data, thus by defining disease severity according to treatment regimens and surgery we aimed to investigate the effect of the longitudinal evolution of disease. We found a significant decrease in the number of OTUs when comparing CD patients with aggressive and non-aggressive disease. There were no changes observed for UC. At the phylum level we found Proteobacteria to be significantly more abundant in CD patients with aggressive disease compared with non-aggressive disease. Wills *et al*. has previously suggested that treatment with thiopurines influence the microbial composition and diversity as they found a significant decrease in diversity among thiopurine users, no such association was found among users of steroids, anti-TNF alpha inhibitors or users of amino salicylates. However, in the multivariate analysis the results did not reach significance. Furthermore, the microbial shifts observed was on an inter-individual level and no significant difference in presence or relative abundance of any particular species or group was detected based on disease activity on a group level neither in the CD nor in the UC patients^42^. A newer prospective study in incident, treatment naïve pediatric IBD patients found that dysbiosis of the gut microbiota persisted after therapy, regardless of treatments and remission status^34^. Yet another study found that the dynamics of the microbiome composition was influenced by changes in medication but weakly correlated to disease activity^33^.

We have not analyzed the influence of different treatment regimens on the gut microbiota as we believe subgroup analyzes will undermine the statistical power of the study due to small samples, however several medications have shown to impact gut microbiota^43^ including IBD specific medications^33^.

It is possible that patients in risk of relapse and thus in need of add-on therapy (medical or surgical) remain in a condition of dysbiosis over time.

In the study by Naftali *et al*., a comparison was made between a cohort of adult CD patients (called the MEIR cohort) and the RISK cohort consisting of treatment-naïve pediatric CD patients showing that the findings of decreased *F. praustnitzii* and increased *Enterobacteriaceae* characterizing ileal CD in the MEIR cohort was not observed in the RISK cohort. This lead to the assumption that microbial dysbiosis may be characteristic of adult onset disease or may develop during years of illness or treatment or both^44^.

Finally, we wished to investigate if disease phenotype influenced the microbiota. Naftali *et al*. showed that the phenotype (ileal, Ileocolonic or colonic disease) of CD determined the clustering of microbiomal taxa regardless of site of biopsy (terminal ileum or colon) or inflammatory state (inflamed or non-inflamed tissue). Furthermore, they found that ileal CD samples were richer in *Escherichia* (Proteobacteria), whereas colon-involving CD had higher levels of *Faecalibacterium* (Firmicutes) and 2 unidentified genera of the *Clostridiales* and *Ruminococceae*^15^. Gevers *et al*. also reported of this clustering of taxa within phenotype regardless of site of biopsy^32^. In the study by Willing, looking at dysbiosis of twins with IBD, more Firmicutes were found in CD patients with colonic disease compared to healthy, whereas patients with ileal disease tended to have fewer. Conversely, ileal CD patients had more Proteobacteria than healthy subjects, whereas this difference was not observed between colonic CD patients and healthy^45^. In addition, we found a significant increase in the abundance of Verrucomicrobia in CD patients with active disease. The impact of these findings are not clear and needs further research^46^.

The major strength of our study is the use of a population of unselected IBD patients from an inception cohort after 7 years of follow-up. The course of the disease on an individual level was thoroughly described by retrospective review of medical records with medical and surgical interventions being registered together with a thorough phenotypic description based on endoscopic and imaging examinations. The patients have, according to the natural history of the disease, within a wide margin, been exposed to different medications and surgery influencing the course of disease on an individual basis leading the cohort in different directions. Nevertheless, we have been able to show similar shifts in bacterial diversity and abundance as previously described in studies of small numbers including highly selected patients. Potential confounders such as diet, Bristol stool score, BMI and medication could influence results, as well as we, unfortunately, were not able to recruit all the patients to the follow-up visit despite vigorous efforts. Due to ethical guidelines in Denmark patients can only be contacted by letter and answer by a return letter, which may influence the reply rate. The study also has several limitations. We do not have baseline stool microbiota data, thus the results of dysbiosis and disease activity is cross sectional. We have no knowledge of stool consistency of the collected fecal samples. This could influence our results^47^. We also do not know whether patients subjective activity scores were due to other symptoms besides active IBD, such as irritable bowel syndrome symptoms, which could influence results^48^. Finally we did not supplement our activity scoring systems with biomarkers of activity such as fecal calprotectin or blood samples like albumin and C-reactive protein. It has been debated whether the sequencing of samples of mucosal versus stool origin impacts results. In the treatment-naïve CD patients in the RISK cohort, the microbiome profiles found in tissue samples, was not found in stool samples^32^. An endoscopic examination was not included in our follow-up visit, thus biopsy sampling was not an option, however, as mentioned in the study by Gevers^32^, it may not be the site of active inflammation that influences the microbial shifts, but the phenotypic appearance. Unfortunately, our study could not support this hypothesis due to low numbers. Larger cohorts of new-onset, treatment-naïve IBD patients have recently been established and shown remarkable results regarding changes in gut microbiome during the first year of disease^34,35^. It will be interesting to follow the long term effects of disease course on the gut microbiota in future follow-up studies of these cohorts.

## Conclusions

In this cohort of unselected IBD patients with 7 years of disease duration, we found shifts in the microbial community of the gut comparable to findings in studies of smaller numbers and highly selected patients. In the cross-sectional design taking disease activity into account, we could only find this change among UC patients. In the longitudinal design, taking the course of disease into account the shift was found in CD patients. We did not find any effect of phenotypic appearance. Our results support the hypothesis that not only disease activity influences the gut microbiota, but also that disease severity and treatment have a forwarded effect on the microbial community of the gut, as changes seem to be more persistent in CD patients with aggressive disease over time. However, it could also be speculated that shifts in the microbiota has a forwarded effect on disease severity.

## Materials and Methods

### Study population and sample collection

In 2003–04, 513 patients (300 UC, 213 CD) were diagnosed with IBD in a well-defined area of Copenhagen, Denmark and data were collected to implement an inception cohort^49^. In 2011–12, data on phenotypic changes, medical treatment and surgery were collected retrospectively by reviewing the medical records. The longitudinal retrospective follow-up did not include subjective data or biomarkers of disease activity. In 2011–12, patients were, by letter, invited to participate in a clinical follow-up visit (patients with mental illness, no Danish language skills, emigration, migration out of study area, wished not to be contacted or lost to follow- up and patients who died during follow-up were not contacted). 140 patients (82 CD, 58 UC) were included in this study. Disease activity was assessed at the follow-up visit by validated scoring systems (Harvey-Bradshaw Index for CD (HB-score) and Simple Clinical Colitis Activity Index (SCCAI) for UC) and smoking status registered. Patients collected a fecal sample at home and sent it by mail to the laboratory where it was stored at minus 80 degrees Celsius until the analyses was performed.

A group of healthy subjects (n = 30) were used as controls. None of the controls had received antibiotics 3 months prior to inclusion. We did not have data on diet, medication or BMI of the controls. As the controls were volunteered co-workers and students, no matching was performed. All the included participants gave informed written consent. Demographic and clinical characteristics of patients and healthy controls are summarized in Table 1. The details with regard to inclusion and exclusion in the follow-up study and disease course have previously been described^36,49^,

**Table 1:** Demographic and clinical characteristics of the 140 IBD patients included and 30 controls in the study.

Characteristics	UC n = 82	CD n = 58	Controls n = 30
Male/female %	35 (43)/47 (57)	24 (41)/ 34 (59)	12 (40)/18 (60)
Age (years), median, (range) at clinical follow-up	39.8 (24.9–81.2)	46.9 (21.0–72.9)	33.0 (18.8–53.9)
**Medication during follow-up**			
5-Aminosalicylic acid/salazopurine (%)	67 (82)	41 (71)	
Azathioprine/6-Mercaptopurine (%)	25 (30)	41 (71)	
Corticosteroids (%)	41 (50)	43 (74)	
Anti-TNF (%)	7 (8)	21 (36)	
Medication at time of follow-up	5 NA	3 NA	
Medication free (%)	20 (24)	20 (34)	
5-Aminosalicylic acid/salazopurine (%)	52 63)	6 (10)	
Azathioprine/6-Mercaptopurine (%)	10 (12)	21 (36)	
Corticosteroids (%)	5 (6)	5 (9)	
Anti-TNF (%)	1 (1)	7 (12)	
Antibiotics 2 months prior to sampling	5 NA	2 NA	
Yes (%)	12 (16)	2 (4)	0 (0)
Disease activity	1 NA		
HB-index <5 (remission) (%)	NA	49 (84)	
HB-index ≥5 (active) (%)	NA	9 (16)	
SCCAI <3 (remission) (%)	59 (73)	NA	
SCCAI ≥3 (active) (%)	22 (27)	NA	
Disease extent (maximum)		NA	
Proctitis E1 (%)	20 (24)	NA	
Left-sided E2 (%)	33 (40)	NA	
Extensive E3 (%)	29 (35)	NA	
Disease localisation (maximum)	NA		
Terminal ileum L1 (%)		5 (9)	
Colon L2 (%)		29 (50)	
Ileocolon L3 (%)		16 (28)	
Upper GI L4 (%)		8 (14)	
Disease behaviour (maximum)	NA		
Inflammatory B1 (%)		36 (62)	
Stricturing B2 (%)		9 (16)	
PenetratingB3 (%)		13 (22)	
**Smoking at diagnosis (%)**			
Current	8 (10)	24 (41)	
Former or never	74 (90)	34 (59)	

N: number, (%): percentage of total, NA: not applicable.

## Definitions

### Disease activity and severity

CD patients were scored with the HBI-score during the follow- up visit, 7 years after inclusion^50^. HBI-score is a simpler score compared to the Crohn’s Disease Activity Index (CDAI) and consists of clinical parameters including: general well-being, abdominal pain, number of liquid stools per day, abdominal mass and extraintestinal manifestations. The HBI-Index provides the categorization of patients into different symptom groups: remission (HBI <5), mild disease (5–7), moderate disease (8–16) and severe disease (>16).

In UC patients, disease activity was assessed by the SCCAI-score questionnaire^51^. SCCAI is a symptom scoring questionnaire, consisting of questions regarding: bowel frequency (day), bowel frequency (night), urgency of defecation, blood in stool, general well-being and extra-intestinal manifestations. Scoring range is between 0 and 19. A SCCAI score of ≤2 was defined as remission, 3–5 as mild disease activity, 6–11 as moderately active disease and ≥12 as severely active disease. A score more than or equal to 3 was defined as active disease.

A severe disease course was defined as ≥3 courses of systemic steroids (≥50 mg/day) and/or biological therapy (any dose) and/or surgical resection (CD patients) during the 7 years of FU.

### IBD phenotype

The disease localization and behavior of CD were described according to the Vienna classification at diagnosis and during follow-up^52^. Maximum extent at follow-up was used in the analyses.

The extent of disease in UC was defined as E1: proctitis (proximal extent to the sigmoid colon), E2: left sided (to the splenic flexure), or E3: extensive disease (beyond the splenic flexure)^53^. Maximum extent at follow-up was used in the analyses.

### Laboratory work

#### DNA extraction

DNA was extracted using the PowerSoil DNA Isolation Kit (MO-BIO), which has previously been found to be well suited for fecal samples^54^. Negative controls were included from DNA extraction^55^. All plates included a mock community to serve as an internal control for calibration of the bioinformatical pipeline.

The 16S rDNA V3-V4 region as targeted using the primers S-D-Bact-0341-b-S-17 and S-D-Bact-0785-a-A-21^56^. These are universal bacterial 16S rDNA primers, which target the V3-V4 region. The following PCR program was used: 98 °C for 30 sec, 25x (98 °C for 10 s, 55 °C for 20 s, 72 °C for 20 s), 72 °C for 5 min. Amplification was verified by running the products on an agarose gel. Indices were added in a subsequent PCR using the Nextera Index Kit V2 (Illumina) with the following PCR program: 98 °C for 30 sec, 8x (98 °C for 10 s, 55 °C for 20 s, 72 °C for 20 s), 72 °C for 5 min. Attachment of barcodes was verified by running the products on an agarose gel.

Products from the nested PCR were pooled and the resulting library cleaned with the Agencourt AMPure XP PCR purification kit (Beckman Coulter). The DNA concentration of pooled libraries was measured on a Qubit fluorometer using the Qubit High Sensitivity Assay Kit (Thermo Fisher Scientific). Sequencing was done on an Illumina MiSeq desktop sequencer using the MiSeq Reagent Kit V3 (Illumina) for 2x 300 bp paired-end sequencing.

### Bioinformatical analysis

The 64-bit versions of USEARCH 8.0.1517^57^ and mothur v.1.35.1^58^ was used in combination with several in-house programs for bioinformatical analysis of the sequence data. Following tag identification and trimming, all sequences from all samples were pooled. Paired end reads were merged, truncating reads at a quality score of 4, requiring at least 100 bp overlap and a merged read length between 300 and 600 bp in length. Sequences with ambiguous bases, without perfect match to the primers, or homopolymer length greater than 8 were discarded and primer sequences trimmed. Reads were quality filtered, discarding reads with more than 5 expected errors and sequences are strictly dereplicated, discarding clusters smaller than 5. Sequences were clustered into OTUs (operational taxonomical units) at 97% sequence similarity, using the most abundant strictly dereplicated reads as centroids and discarding suspected chimeras based on internal comparison. Additional suspected chimeric OTUs were discarded based on comparison with the Ribosomal Database Project classifier training set v9^59^ using UCHIME^60^. Taxonomic assignment of OTUs was done using the method by Wang *et al*.^61^ with mothur’s PDS version of the RDP training database v14. Following this, samples were rarified to the lowest sequence number found in a sample (2780).

### Statistics

Statistical analyses were done in R. The data are given in numbers and percentages or median and ranges. Mann-Whitney U tests and Kruskal-Wallis tests were used for testing for significant differences between groups.

### Ethical considerations

All research was performed in accordance with hospital guidelines, and informed consent was obtained from all participants. The Regional Ethics Committee (The Capitol Region of Denmark) approved this study (H-1-2011-088) and permission was obtained from the Danish Data Registry (01769 HVH-2012-027).

## Data Availability

The datasets generated during and/or analysed during the current study are available from the corresponding author on reasonable request.

## References

[1] Wallace KL, Zheng L-B, Kanazawa Y, Shih DQ, Wi- F (2014). Immunopathology of inflammatory bowel disease. World J Gastroenterol.

[2] Frank DN (2007). Molecular-phylogenetic characterization of microbial community imbalances in human inflammatory bowel diseases. Proc. Natl. Acad. Sci. USA.

[3] Sokol H (2006). Specificities of the fecal microbiota in inflammatory bowel disease. Inflamm. Bowel Dis..

[4] Peterson AM (2007). A checklist for medication compliance and persistence studies using retrospective databases. Value.Health.

[5] Ott SJ (2004). Reduction in diversity of the colonic mucosa associated bacterial microflora in patients with active inflammatory bowel disease. Gut.

[6] Lowenberg M, Peppelenbosch M, Hommes D (2006). Biological therapy in the management of recent-onset Crohn’s disease: why, when and how?. Drugs.

[7] Manichanh C (2006). Reduced diversity of faecal microbiota in Crohn’s disease revealed by a metagenomic approach. Gut.

[8] Baumgart M (2007). Culture independent analysis of ileal mucosa reveals a selective increase in invasive *Escherichia coli* of novel phylogeny relative to depletion of Clostridiales in Crohn’s disease involving the ileum. ISME J..

[9] Gophna U, Sommerfeld K, Gophna S, Doolittle WF, Veldhuyzen Van Zanten SJO (2006). Differences between tissue-associated intestinal microfloras of patients with Crohn’s disease and ulcerative colitis. J. Clin. Microbiol..

[10] Rajca S (2014). Alterations in the intestinal microbiome (dysbiosis) as a predictor of relapse after infliximab withdrawal in Crohn’s disease. Inflamm. Bowel Dis..

[11] Lupp C (2007). Host-Mediated Inflammation Disrupts the Intestinal Microbiota and Promotes the Overgrowth of Enterobacteriaceae. Cell Host Microbe.

[12] Sokol H (2008). Faecalibacterium prausnitzii is an anti-inflammatory commensal bacterium identified by gut microbiota analysis of Crohn disease patients. Proc. Natl. Acad. Sci. USA.

[13] Martinez-Medina M, Aldeguer X, Gonzalez-Huix F, Acero D, Garcia-Gil LJ (2006). Abnormal microbiota composition in the ileocolonic mucosa of Crohn’s disease patients as revealed by polymerase chain reaction-denaturing gradient gel electrophoresis. Inflamm. Bowel Dis..

[14] Swidsinski Alexander, Loening-Baucke Vera, Vaneechoutte Mario, Doerffel Yvonne (2008). Active Crohnʼs disease and ulcerative colitis can be specifically diagnosed and monitored based on the biostructure of the fecal flora. Inflammatory Bowel Diseases.

[15] Naftali T (2016). Distinct Microbiotas are Associated with Ileum-Restricted and Colon-Involving Crohn’s Disease. Inflamm. Bowel Dis..

[16] Martinez-Medina Margarita, Aldeguer Xavier, Lopez-Siles Mireia, González-Huix Ferran, López-Oliu Carles, Dahbi Ghizlane, Blanco Jesus E., Blanco Jorge, Garcia-Gil Jesus L., Darfeuille-Michaud Arlette (2009). Molecular diversity of Escherichia coli in the human gut: New ecological evidence supporting the role of adherent-invasive E. coli (AIEC) in Crohnʼs disease. Inflammatory Bowel Diseases.

[17] Willing B (2009). Twin studies reveal specific imbalances in the mucosa-associated microbiota of patients with ileal Crohn’s disease. Inflamm. Bowel Dis..

[18] Darfeuille-Michaud A (2004). High prevalence of adherent-invasive *Escherichia coli* associated with ileal mucosa in Crohn’s disease. Gastroenterology.

[19] Sokol H (2009). Low counts of Faecalibacterium prausnitzii in colitis microbiota. Inflamm. Bowel Dis..

[20] Vermeiren J (2012). Decreased colonization of fecal Clostridium coccoides/Eubacterium rectale species from ulcerative colitis patients in an *in vitro* dynamic gut model with mucin environment. FEMS Microbiol. Ecol..

[21] Morgan XC (2012). Dysfunction of the intestinal microbiome in inflammatory bowel disease and treatment. Genome Biol..

[22] Nagalingam NA, Lynch SV (2012). Role of the microbiota in inflammatory bowel diseases. Inflammatory Bowel Diseases.

[23] Mirsepasi-Lauridsen HC (2016). Extraintestinal pathogenic *Escherichia coli* are associated with intestinal inflammation in patients with ulcerative colitis. Sci. Rep..

[24] Mirsepasi-Lauridsen HC (2016). Secretion of Alpha-Hemolysin by *Escherichia coli* Disrupts Tight Junctions in Ulcerative Colitis Patients. Clin. Transl. Gastroenterol..

[25] Vejborg RM, Hancock V, Petersen AM, Krogfelt KA, Klemm P (2011). Comparative genomics of *Escherichia coli* isolated from patients with inflammatory bowel disease. BMC Genomics.

[26] Costello SP (2017). Systematic review with meta-analysis: faecal microbiota transplantation for the induction of remission for active ulcerative colitis. Aliment. Pharmacol. Ther..

[27] Ramadas AV, Gunesh S, Thomas GAO, Williams GT, Hawthorne AB (2010). Natural history of Crohn’s disease in a population-based cohort from Cardiff (1986-2003): a study of changes in medical treatment and surgical resection rates. Gut.

[28] Vester-Andersen M (2014). Hospitalisation, surgical and medical recurrence rates in inflammatory bowel disease 2003-2011—a Danish population-based cohort study. J. Crohns. Colitis Dec 1.

[29] Henriksen M (2007). Clinical course in Crohn’s disease: results of a five-year population-based follow-up study (the IBSEN study). Scand.J.Gastroenterol..

[30] Cleynen I (2016). Inherited determinants of Crohn’s disease and ulcerative colitis phenotypes: a genetic association study. Lancet (London, England).

[31] Knights D (2014). Complex host genetics influence the microbiome in inflammatory bowel disease. Genome Med..

[32] Gevers D (2014). The treatment-naive microbiome in new-onset Crohn’s disease. Cell Host Microbe.

[33] Halfvarson J (2017). Dynamics of the human gut microbiome in inflammatory bowel disease. Nat. Microbiol..

[34] Olbjørn C (2019). Fecal microbiota profiles in treatment-naïve pediatric inflammatory bowel disease – associations with disease phenotype, treatment, and outcome. Clin. Exp. Gastroenterol..

[35] Schirmer M (2018). Compositional and Temporal Changes in the Gut Microbiome of Pediatric Ulcerative Colitis Patients Are Linked to Disease Course. Cell Host Microbe.

[36] Vester-Andersen MK (2014). Disease course and surgery rates in inflammatory bowel disease: a population-based, 7-year follow-up study in the era of immunomodulating therapy. Am. J. Gastroenterol..

[37] Fujimoto T (2013). Decreased abundance of Faecalibacterium prausnitzii in the gut microbiota of Crohn’s disease. J. Gastroenterol. Hepatol..

[38] Machiels K (2014). A decrease of the butyrate-producing species Roseburia hominis and Faecalibacterium prausnitzii defines dysbiosis in patients with ulcerative colitis. Gut.

[39] Varela E., Manichanh C., Gallart M., Torrejón A., Borruel N., Casellas F., Guarner F., Antolin M. (2013). Colonisation byFaecalibacterium prausnitziiand maintenance of clinical remission in patients with ulcerative colitis. Alimentary Pharmacology & Therapeutics.

[40] Prosberg M, Bendtsen F, Vind I, Petersen AM, Gluud LL (2016). The association between the gut microbiota and the inflammatory bowel disease activity: a systematic review and meta-analysis. Scand. J. Gastroenterol..

[41] Walujkar SA (2014). Characterization of bacterial community shift in human Ulcerative Colitis patients revealed by Illumina based 16S rRNA gene amplicon sequencing. Gut Pathog..

[42] Wills ES (2014). Fecal microbial composition of ulcerative colitis and Crohn’s disease patients in remission and subsequent exacerbation. PLoS One.

[43] Maier L (2018). Extensive impact of non-antibiotic drugs on human gut bacteria. Nature.

[44] Naftali T (2016). Distinct Microbiotas are Associated with Ileum-Restricted and Colon-Involving Crohnʼs Disease. Inflamm. Bowel Dis..

[45] Willing BP (2010). A pyrosequencing study in twins shows that gastrointestinal microbial profiles vary with inflammatory bowel disease phenotypes. Gastroenterology.

[46] Derrien M, Belzer C, de Vos WM (2017). Akkermansia muciniphila and its role in regulating host functions. Microb. Pathog..

[47] Vandeputte D (2016). Stool consistency is strongly associated with gut microbiota richness and composition, enterotypes and bacterial growth rates. Gut.

[48] Spiller R, Major G (2016). IBS and IBD — separate entities or on a spectrum?. Nat. Rev. Gastroenterol. Hepatol..

[49] Vind I (2006). Increasing incidences of inflammatory bowel disease and decreasing surgery rates in Copenhagen City and County, 2003-2005: a population-based study from the Danish Crohn colitis database. Am.J.Gastroenterol..

[50] Harvey RF, Bradshaw JM (1980). A simple index of Crohn’s-disease activity. Lancet (London, England).

[51] Walmsley RS, Ayres RC, Pounder RE, Allan RN (1998). A simple clinical colitis activity index. Gut.

[52] Gasche C (2000). A simple classification of Crohn’s disease: report of the Working Party for the World Congresses of Gastroenterology, Vienna 1998. Inflamm. Bowel Dis..

[53] Satsangi J, Silverberg MS, Vermeire S, Colombel JF (2006). The Montreal classification of inflammatory bowel disease: controversies, consensus, and implications. Gut.

[54] Aagaard K (2013). The Human Microbiome Project strategy for comprehensive sampling of the human microbiome and why it matters. FASEB J..

[55] Weiss S (2014). Tracking down the sources of experimental contamination in microbiome studies. Genome Biol..

[56] Klindworth A (2013). Evaluation of general 16S ribosomal RNA gene PCR primers for classical and next-generation sequencing-based diversity studies. Nucleic Acids Res..

[57] Edgar RC (2013). UPARSE: highly accurate OTU sequences from microbial amplicon reads. Nat. Methods.

[58] Schloss PD (2009). Introducing mothur: open-source, platform-independent, community-supported software for describing and comparing microbial communities. Appl. Environ. Microbiol..

[59] Oussalah A (2010). A multicenter experience with infliximab for ulcerative colitis: outcomes and predictors of response, optimization, colectomy, and hospitalization. Am. J. Gastroenterol..

[60] Edgar RC, Haas BJ, Clemente JC, Quince C, Knight R (2011). UCHIME improves sensitivity and speed of chimera detection. Bioinformatics.

[61] Wang Q, Garrity GM, Tiedje JM, Cole JR (2007). Naive Bayesian classifier for rapid assignment of rRNA sequences into the new bacterial taxonomy. Appl. Environ. Microbiol..

